# Does More Flexible Pricing Always Pay? Profit-Driven Pricing and Market Stability Under Platform Regulation

**DOI:** 10.3390/e28050571

**Published:** 2026-05-19

**Authors:** Le-Bin Wang, Jian Chai, Ying Yang

**Affiliations:** School of Economics and Management, Xidian University, Xi’an 710126, China

**Keywords:** price competition, dynamic complexity, platform regulation, chaos, entropy

## Abstract

This paper studies a dynamic price adjustment system in platform markets, where sellers continuously revise prices, and examines its implications for market stability. We develop a platform-led discrete-time Stackelberg game model to describe the evolution of sellers’ prices and price adjustment speeds under bounded rationality. Unlike previous studies that treat adjustment speed as exogenous, we model it as an endogenous state variable shaped by profit incentives, behavioral inertia, and price fluctuations. We derive the interior symmetric equilibrium and show that profit-driven acceleration increases sellers’ adjustment speed. When this speed exceeds the stability threshold, the system may leave the stable region, causing bifurcations and complex dynamics. We then introduce a platform-imposed upper bound on adjustment speeds and demonstrate that appropriate regulation can restore stability while balancing market responsiveness and efficiency. Numerical simulations illustrate that moderate acceleration improves profitability, whereas excessive acceleration can lead to low-profit regimes. Entropy-based metrics are used to quantify system complexity, and an entropy-triggered feedback-control mechanism is proposed to mitigate excessive volatility while maintaining flexibility. Overall, the study highlights the importance of governing adjustment dynamics rather than solely focusing on price levels.

## 1. Introduction

In modern e-commerce platforms and sharing economy markets, dynamic pricing has become a core strategy through which platforms and sellers compete [1]. By adjusting prices in response to demand fluctuations, inventory changes, and competitors’ actions, firms seek to maximize transaction volume and profitability. In practice, sellers increasingly rely on real-time pricing systems to respond quickly to changing market conditions. For example, pricing systems used by Amazon sellers and surge-pricing strategies adopted by Uber both rely on flexible price adjustments to improve profitability for platforms and sellers alike [2].

Despite the short-term profitability gains brought by flexible pricing, determining the appropriate speed of price adjustment remains an important challenge. In practice, sellers often treat adjustment speed as a strategic decision variable and determine it endogenously. Faster adjustment enables sellers to respond more rapidly to demand changes and competitors’ pricing actions, thereby capturing short-term profit opportunities. However, excessively rapid adjustment may amplify price fluctuations and destabilize the market, ultimately harming long-run profitability [3,4]. As a result, sellers face a fundamental trade-off between flexibility and stability.

At the same time, platforms, as rule setters in the market, often impose constraints on price adjustment speeds to control market volatility [5]. The upper bounds on adjustment speeds affect sellers’ pricing behavior and reflect the platform’s trade-off between market stability and competitiveness. However, such regulatory interventions are not without cost. Loose regulation may lead to excessive competition and heightened price volatility, whereas overly stringent regulation may suppress market vitality, reduce sellers’ incentives to compete, and even diminish overall platform revenue. Consequently, platforms face a similar trade-off problem: how to maintain market stability while preserving competition and improving social welfare through appropriate regulatory intensity.

Although existing studies have examined dynamic pricing, platform governance, duopoly competition, and complex systems from various perspectives [3,4,5,6,7,8,9,10], several important gaps remain. First, most dynamic pricing studies treat adjustment speed as an exogenous parameter and pay limited attention to why sellers endogenously increase pricing aggressiveness in response to profit incentives and market fluctuations [6,7]. Second, platform-governance research mainly focuses on commissions, ranking algorithms, information disclosure, and pricing authority, while paying insufficient attention to whether platforms should regulate sellers’ price adjustment speeds [5]. Third, although the bounded-rationality and complex-systems literature has extensively analyzed local stability, bifurcation, and nonlinear dynamics in duopoly and supply-chain systems [3,4,7,9,10], the adjustment process itself is usually treated as exogenously given. Existing studies rarely integrate endogenous adjustment speed, platform regulation, and adaptive control mechanisms into a unified analytical framework. Consequently, the literature still provides limited understanding of how profit-driven acceleration affects market stability, system complexity, and welfare outcomes in platform markets.

To address these gaps, this paper develops a platform-led discrete dynamic pricing model by integrating a Stackelberg game framework with bounded-rational adjustment mechanisms. In the model, sellers’ adjustment speeds evolve endogenously according to profit incentives, price fluctuations, and behavioral inertia. We first analyze how endogenous adjustment speed affects local stability and market dynamics. We then introduce platform-imposed upper bounds on adjustment speeds and examine whether regulation can restore stability and improve platform performance or social welfare. Finally, we incorporate entropy-based complexity measures and entropy-triggered feedback control mechanisms to evaluate how adaptive regulation can mitigate excessive volatility and stabilize the pricing system. Robustness analyses under alternative competition and commission settings further confirm the stability of the main findings.

Different from the existing discrete-time Stackelberg pricing literature [3], this paper treats adjustment speed itself as an endogenous strategic state variable jointly shaped by profit incentives, behavioral inertia, and platform regulation. Existing studies typically regard adjustment speed as exogenously given and mainly focus on how fixed adjustment parameters affect system stability. In contrast, this paper explains how profit-driven acceleration may destabilize market dynamics and how platforms can govern adjustment behavior through speed caps and adaptive feedback control. These findings suggest that platform governance in algorithmic pricing environments should focus not only on pricing outcomes, but also on the dynamics of the pricing-adjustment process itself.

The remainder of the paper is organized as follows. Section 2 reviews the related literature. Section 3 presents the model setup and dynamic adjustment mechanism, and develops the platform-regulated duopoly game. Section 4 derives the interior symmetric equilibrium and local stability conditions, analyzes how platform regulation restores stability. Section 5 provides numerical simulations to illustrate the effects of different regulatory intensities on profits and market stability, and proposes a chaos control mechanism for the platform. Section 6 concludes with key findings, managerial implications, and directions for future research.

## 2. Literature Review

Research related to this paper can be broadly classified into four streams. The first focuses on dynamic pricing and algorithmic pricing, examining how firms update prices based on demand, reviews, inventory, and consumer behavior. The second concerns platform governance and price control, emphasizing the role of platforms in price setting, allocation of pricing authority, and rule design. The third investigates bounded rationality and complex dynamics, focusing on local stability, bifurcation, and discrete-time nonlinear behavior under adaptive pricing mechanisms. The fourth explores chaos control in economic systems, analyzing how feedback control, delay control, or parameter regulation can restore stability. Existing studies differ in their treatment of adjustment dynamics, platform intervention, and system complexity. Comparatively little attention has been paid to how endogenous adjustment speed, platform regulation, entropy-based complexity, and adaptive feedback control interact within a unified platform-pricing framework.

### 2.1. Dynamic Pricing and Algorithmic Pricing

The dynamic pricing literature primarily examines how firms update prices in response to continuously evolving market environments, incorporating information such as demand, inventory, reviews, consumer learning, and reference prices. Kopalle et al. [1] provide a comprehensive overview of the fundamentals, managerial implications, and future research directions of dynamic pricing. Shin et al. [6] study how online reviews influence consumer learning processes and, in turn, affect firms’ dynamic pricing decisions. Chung et al. [11] extend the analysis by incorporating reward point redemption mechanisms, showing that when intertemporal redemption behavior is considered, optimal pricing paths are jointly shaped by incentive schemes and demand shifting over time. Abhishek et al. [12], from an online resource allocation perspective, examine the joint determination of pricing and timing decisions, highlighting that pricing strategies often co-evolve with resource allocation and transaction timing in dynamic environments.

Recent algorithmic pricing studies explore consumer responses and implementation boundaries, including reference prices, trust, fairness perceptions, and search behavior. Prakash and Spann [13] show that price change magnitude and frequency influence consumers’ reference prices. Vomberg et al. [14] find algorithmic pricing can reduce consumer trust and intensify search behavior. Spann et al. [15] discuss the marketing logic and regulatory implications of algorithmic pricing. Wamsler et al. [16] examine whether firms should retain or delegate pricing authority, Lu and Wang [17] introduce social learning into new product pricing, and Syed et al. [18] analyze data-driven pricing for perishable goods. Overall, this stream highlights the importance of dynamic pricing for revenue management and market responsiveness, while also identifying practical limitations in digital retail.

Beyond consumer responses, recent research examines strategic interactions among algorithmic pricing agents. Calvano et al. [19] demonstrate that reinforcement-learning pricing agents may tacitly coordinate in repeated pricing games autonomously, without explicit communication. Miklós-Thal and Tucker [20] show that improved algorithmic demand prediction can help tailor prices but also increases incentives to undercut in high-demand periods, potentially undermining collusion. Asker et al. [21] show that different algorithmic learning protocols can generate markedly different pricing outcomes, highlighting the role of feedback and learning mechanisms.

Despite these advances, important limitations remain in the existing dynamic- and algorithmic-pricing literature. Most studies focus on pricing outcomes, demand learning, or collusive behavior among algorithmic agents [19,20,21]. They generally treat adjustment intensity, learning frequency, or pricing aggressiveness as exogenous, rather than evolving strategically through market feedback. Studies on consumer responses [13,14] mainly examine fairness perception or search behavior, with little attention to how adjustment speed itself evolves. In contrast, the present paper models adjustment speed as an endogenous variable driven by profit incentives, price fluctuations, and behavioral inertia, shifting the focus from static pricing outcomes to the evolution of pricing aggressiveness and its impact on market stability and system complexity.

### 2.2. Platform Governance, Price Control, and Rule Design

The second stream focuses on platform governance. Platforms are not just intermediaries; they also shape market efficiency and competition via commission schemes, allocation of control rights, user interventions, and pricing rules. Cachon et al. [5] analyze price control and regulation, showing that allocation of pricing authority affects surplus distribution. Filippas et al. [22] show that centralized pricing improves asset utilization but increases transaction costs, while partial user control balances efficiency and flexibility. Xu et al. [23] demonstrate that aggressive surge pricing increases complaints, whereas price caps mitigate these effects. Dogan and Jacquillat [24] examine collective dynamic pricing in on-demand platforms, while Dong et al. [25], Xu et al. [26], Yang et al. [27], and Zhuo et al. [28] study platform-specific pricing strategies. These studies emphasize pricing outcomes and authority, but pay limited attention to whether platforms should govern speed, frequency, or aggressiveness of algorithmic price updates. The present paper treats adjustment speed as a core governance object, analyzing how platform-imposed speed regulation affects system stability, entropy growth, market complexity, and welfare.

Beyond platform-specific settings, other regulatory studies also indicate that external policy tools significantly influence firms’ pricing and strategic behavior. For example, Ji et al. [29] study pricing and emission reduction decisions under carbon regulation and show that regulatory environments systematically alter firm strategies and market outcomes. This suggests that extending regulatory focus to price adjustment speed in platform markets is theoretically well grounded.

Existing platform-governance studies mainly examine pricing authority allocation, surge-pricing rules, price caps, and platform coordination mechanisms. For example, Cachon et al. [5] and Filippas et al. [22] focus on how centralized pricing and control-right allocation influence efficiency and surplus distribution, while Xu et al. [23] emphasize the role of price caps in mitigating consumer complaints generated by aggressive pricing strategies. Similarly, Dogan and Jacquillat [24] analyze collective pricing coordination in platform environments. However, these studies primarily regulate pricing outcomes or pricing authority, rather than the dynamics of the pricing-adjustment process itself. In particular, relatively little attention has been paid to whether platforms should govern the speed, frequency, or aggressiveness of algorithmic price updates. Compared with this literature, the present paper treats adjustment speed as a core governance object and analyzes how platform-imposed speed regulation affects system stability, entropy growth, market complexity, platform performance, and welfare outcomes.

### 2.3. Bounded Rationality, Discrete Dynamic Games, and Complex Systems

The third stream introduces bounded rationality into oligopoly competition, platform competition, and supply chain games, leading to a growing literature on complex dynamics. Foundational studies on boundedly rational oligopoly dynamics have shown that iterative price or quantity adjustment processes can generate local instability, bifurcations, and chaotic behavior in discrete-time competitive systems. For example, Kopel [30] analyzes simple and complex adjustment dynamics in Cournot duopoly models, while Bischi and Naimzada [31] provide a global analysis of dynamic duopoly games with bounded rationality. Building on these foundational contributions, subsequent studies typically assume that decision makers do not solve fully rational static equilibria but instead adjust prices, quantities, or investments incrementally based on marginal profits, market feedback, or gradient-based rules, resulting in discrete-time nonlinear dynamic systems. Xiao et al. [3] analyze dynamic behavior in a differentiated product Stackelberg model under bounded rationality and show that parameter changes can lead to loss of local stability and complex bifurcations. Zhu et al. [4] study low-carbon supply chain dynamics and demonstrate that strategy evolution exhibits rich nonlinear patterns under environmental policies. Mai et al. [7] examine pricing in a book supply chain and find that agency fees and adjustment speeds significantly influence system complexity. Similarly, Liu et al. [9] and Chen et al. [10] analyze green supply chains and show that parameter variations can induce bifurcation and chaos. More recent studies extend these analyses to specific contexts: Huang et al. [32] document complex dynamics in low-carbon maritime supply chains; Wang et al. [33] analyze fractal basin structures in supply chain competition; and Xie et al. [34] investigate price stability in blockchain-enabled cross-border dual-channel supply chains.

Bounded rationality introduces rich dynamics in oligopoly, platform competition, and supply chains. Kopel [30] analyzes simple and complex adjustment dynamics in Cournot duopoly, Bischi and Naimzada [31] provide global analysis of dynamic duopoly games, and Xiao et al. [3], Zhu et al. [4], Mai et al. [7], Liu et al. [9], Chen et al. [10] extend these results to platform and supply-chain settings. Prior work demonstrates that exogenously specified adjustment parameters can induce instability and complex patterns. However, these studies rarely address why adjustment aggressiveness evolves endogenously or how platform governance can moderate it. Although some use entropy to describe system complexity [35], it is typically not integrated into adaptive control. The present paper models adjustment speed as an endogenous, profit-driven state variable and integrates entropy-based complexity analysis with platform regulation and adaptive feedback control.

### 2.4. Chaos Control and Stability Restoration in Economic Systems

The fourth stream examines how to control economic systems that exhibit complex or chaotic dynamics. Early studies demonstrate that appropriate feedback control, delay control, and parameter regulation can effectively suppress nonlinear instability. In recent years, such approaches have increasingly been applied to supply chains and platform competition settings. Chen and Zhou [8] introduce feedback control into an omnichannel supply chain model and show that regulating key decision variables can reduce system complexity and restore stability. Building on this, Wang et al. [33] characterize dynamic evolution across parameter regions using fractal basin structures, providing important insights for chaos control. Xie et al. [34] further analyze stability in blockchain-enabled cross-border supply chains and demonstrate that adjusting key parameters can shrink chaotic regions and indirectly stabilize the system.

At the same time, with the widespread adoption of algorithmic pricing in platform markets, some studies begin to examine the externalities of price fluctuations from the perspectives of algorithmic governance and consumer behavior. Choi et al. [36] and Wang et al. [37] show that algorithmic pricing may intensify perceptions of price unfairness and trigger negative behavioral responses. This suggests that overly aggressive dynamic pricing may generate not only system-level instability but also behavioral externalities, reinforcing the need to regulate price dynamics.

Chaos-control studies examine stabilization of nonlinear systems. Chen and Zhou [8] introduce feedback control in supply chains. Wang et al. [33] characterize parameter regions using fractal basins, and Xie et al. [34] analyze stability in blockchain-enabled supply chains. Choi et al. [36] and Wang et al. [37] show that algorithmic pricing can trigger unfairness perceptions and behavioral responses. However, these studies rarely integrate endogenous adjustment speed, platform regulation, entropy-based complexity, and adaptive control into a single framework. The present paper develops an entropy-triggered adaptive control mechanism that regulates excessive adjustment-speed acceleration, restoring stability without constraining price levels directly.

### 2.5. Positioning and Contributions

Taken together, the existing literature has generated important insights into dynamic pricing, platform governance, bounded-rationality dynamics, and chaos control [1,5,8,19,20,21,22,23,24,25,26,27,28,29,30,31,32,33,34,35,36,37]. However, these streams differ substantially in analytical focus. Dynamic-pricing and algorithmic-pricing studies mainly examine pricing outcomes, consumer responses, demand learning, and algorithmic interaction [1,13,14,15,16,17,18,19,20,21], while platform-governance research focuses primarily on pricing authority, price caps, and coordination mechanisms [5,22,23,24,25,26,27,28,29]. By contrast, the bounded-rationality and complex-systems literature emphasizes bifurcation and chaos under exogenously specified adjustment rules [3,4,7,9,10,30,31,32,33,34,35], and chaos-control studies mainly investigate generic stabilization mechanisms in nonlinear economic systems [8,33,34]. Comparatively little attention has been paid to how endogenous adjustment-speed dynamics, platform regulation, entropy-based complexity, and adaptive control interact within a unified platform-pricing framework.

To address these gaps, this paper develops a platform-led discrete-time dynamic pricing model in which sellers’ adjustment speeds evolve endogenously under profit incentives, behavioral inertia, and market fluctuations. Unlike existing studies that mainly regulate pricing outcomes or assume exogenous adjustment processes [5,19,22,30], we treat adjustment speed itself as a core governance object and analyze how platform-imposed speed regulation affects system stability, entropy growth, market complexity, and welfare outcomes. Furthermore, compared with existing chaos-control studies [8,33,34], this paper develops an entropy-triggered adaptive feedback-control mechanism that restores stability while preserving pricing flexibility. In this way, the paper integrates endogenous adjustment dynamics, platform governance, entropy-based complexity analysis, and adaptive chaos control into a unified framework for algorithmic pricing systems.

## 3. Problem Description and Model Setup

**Figure 1:**
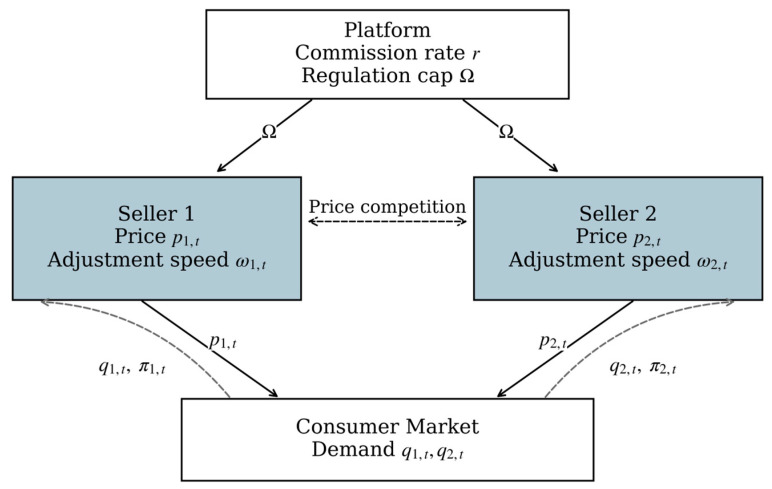
Platform–Seller Interaction Structure.

In the platform market studied here, a clear hierarchical structure exists between the platform and the sellers. As the market rule-setter, the platform first sets an upper bound on sellers’ price adjustment speeds. Given this constraint, two sellers offering substitutable products then compete on prices and dynamically adjust both their prices and adjustment speeds based on current profits and market feedback. This interaction can be modeled as a Stackelberg leader–follower game. Recognizing that sellers do not instantaneously achieve fully rational optimal decisions but adjust strategies iteratively in a boundedly rational manner, we develop a discrete-time dynamic competition model within this Stackelberg framework, as shown in Figure 1.

The platform’s commission rate is treated as exogenous, reflecting pre-existing pricing rules. In contrast, the regulatory upper bound on price adjustment speeds is the key policy instrument the platform can actively adjust in the short run. Based on this setup, we next formulate sellers’ demand functions, the profit functions of all participants, and the discrete-time dynamic model describing the evolution of prices and adjustment speeds under platform regulation.

The variables and symbols used in the model are shown in Table 1.

### 3.1. Demand Function and Consumer Surplus

Consider a market consisting of two sellers (indexed by $i\in \left\{1,2\right\}$) and a platform. Let $p_{i,t}$ denote the selling price of seller i at time t. The two sellers offer substitutable products, and the market demand is assumed to be linear. Accordingly, the demand functions faced by the two sellers can be expressed as:

(1) $$q_{1,t}=a-bp_{1,t}+dp_{2,t}$$

(2) $$q_{2,t}=a-bp_{2,t}+dp_{1,t}$$

Here, a>0 represents the baseline market demand, b>0 measures the sensitivity of demand to a seller’s own price, and d∈(0,b) captures the degree of substitutability between the two products. To ensure the well-behavedness of the demand system, we assume that d<b, that is, the negative effect of a seller’s own price on demand is stronger than the positive effect of the rival’s price. The cross-price terms thus capture the competitive interaction between the two sellers.

Combining Equations (1) and (2), the inverse demand functions can be obtained as: $p_{1,t}=a_{0}-b_{0}q_{1,t}-g_{0}q_{2,t}$, $p_{2,t}=a_{0}-b_{0}q_{2,t}-g_{0}q_{1,t}$, where $a_{0}=\frac{(b+d)a}{b^{2}-d^{2}}$, $b_{0}=\frac{b}{b^{2}-d^{2}}$, $g_{0}=\frac{d}{b^{2}-d^{2}}$.

To provide a micro-foundation for the above demand structure, this paper introduces a standard quasi-linear quadratic utility function [38,39]:

(3) $$U(q_{1,t},q_{2,t},m)=m+a_{0}(q_{1,t}+q_{2,t})-\frac{b_{0}}{2}(q_{1,t}^{2}+q_{2,t}^{2})-g_{0}q_{1,t}q_{2,t}$$

Here, m denotes the numeraire good (i.e., residual income), $a_{0}$ represents the baseline utility level, $b_{0}>0$ captures the degree of diminishing marginal utility from own consumption, and $g_{0}$ characterizes the interaction between the two products (i.e., substitutability or complementarity). Under this specification, the consumer’s marginal utilities satisfy: $\frac{\partial U}{\partial q_{1,t}}=a_{0}-b_{0}q_{1,t}-g_{0}q_{2,t}$, $\frac{\partial U}{\partial q_{2,t}}=a_{0}-b_{0}q_{2,t}-g_{0}q_{1,t}$, which are consistent with the inverse demand functions.

Under quasi-linear preferences, consumer surplus is equal to total utility minus total expenditure. Therefore, at time t, we have: $CS_{t}=U(q_{1,t},q_{2,t},m)-m-p_{1,t}q_{1,t}-p_{2,t}q_{2,t}$. Further simplification yields the consumer surplus as:

(4) $$CS_{t}=\frac{b(q_{1,t}^{2}+q_{2,t}^{2})+2dq_{1.t}q_{2.t}}{2(b^{2}-d^{2})}$$

### 3.2. Profit Function and Social Welfare

The platform applies an ad valorem commission scheme, charging a proportion r∈(0,1) of each seller’s sales revenue as commission. Each seller also incurs a constant unit production cost c per unit produced. Under these assumptions, the profit of seller i at time t is given by:

(5) $$\pi_{i,t}=\left(\left(1-r\right)p_{i,t}-c\right)q_{i,t},i=1,2$$

The platform collects commission revenue from the sales of both sellers while incurring a certain regulatory cost. Let $\Omega \in [0,\Omega^{\max}]$ denote the platform’s regulatory intensity over sellers’ price adjustment speeds, that is, the upper bound imposed by the platform on the sellers’ price adjustment speeds. A larger Ω indicates a looser regulatory environment, whereas a smaller Ω implies stricter regulation. Similar to existing literature [40,41], we assume that the regulatory cost of the platform is a quadratic function:

(6) $$C\left(\Omega \right)=\frac{k}{2}{\left(\Omega^{\max}-\Omega \right)}^{2}$$

Here, k>0 is the regulatory cost coefficient. This specification implies that stricter regulation entails higher monitoring and enforcement costs.

Accordingly, the platform’s profit at time t is given by:

(7) $$\Pi_{t}=r\left(p_{1,t}q_{1,t}+p_{2,t}q_{2,t}\right)-C\left(\Omega \right)$$

In addition, from the perspective of social welfare, commission revenue constitutes a transfer payment between sellers and the platform and therefore should not be double-counted. Moreover, dynamic pricing regulation may affect welfare through price stability, as excessive intertemporal price fluctuations can reduce market predictability and weaken consumer confidence. This is consistent with recent evidence that rate-stability regulation in insurance markets changes welfare by altering the trade-off between price stability and market outcomes [42]. To capture this volatility externality in a parsimonious way, we define social welfare at time t as:

(8) $$W_{t}=\left(p_{1,t}-c\right)q_{1,t}+\left(p_{2,t}-c\right)q_{2,t}+CS_{t}-C\left(\Omega \right)-\phi V_{t}$$

Here, $V_{t}={\left(p_{1,t}-p_{1,t-1}\right)}^{2}+{\left(p_{2,t}-p_{2,t-1}\right)}^{2}$ measures the intensity of price volatility, and ϕ>0 captures the social cost of excessive price fluctuations. The quadratic form is used as a reduced-form penalty for instability and volatility rather than as a structural estimate of consumer utility loss [43]. At the symmetric equilibrium, $p_{1,t}=p_{1,t-1}$, so $V_{t}$ equals zero and does not affect the equilibrium social welfare.

### 3.3. Discrete Dynamics Model Construction

For seller 1, the derivative of marginal profit with respect to price is given by:

(9) $$\frac{\partial \pi_{1,t}}{\partial p_{1,t}}=(1-r)a+bc+(1-r)dp_{2,t}-2b(1-r)p_{1,t}$$

Similarly, for seller 2, the derivative of marginal profit with respect to price is given by:

(10) $$\frac{\partial \pi_{2,t}}{\partial p_{2,t}}=(1-r)a+bc+(1-r)dp_{1,t}-2b(1-r)p_{2,t}$$

In platform markets, sellers typically do not solve a fully rational intertemporal optimization problem in each period. Instead, they adjust prices gradually based on current marginal profits. This behavioral rule is consistent with the bounded rationality assumption widely adopted in discrete-time dynamic duopoly models. Let $\omega_{i,t}$ denote the price adjustment speed of seller i at time t. Then, the price of seller i at time t evolves according to the following rule:

(11) $$p_{i,t+1}=p_{i,t}+\omega_{i,t}\frac{\partial \pi_{i,t}}{\partial p_{i,t}},i=1,2$$

Unlike the existing literature, which typically treats $\omega_{i,t}$ as an exogenous parameter, this paper assumes that the price adjustment speed itself is an endogenous variable that evolves over time. Specifically, when current profits are relatively high, sellers tend to increase their adjustment speed, reflecting greater confidence and more aggressive pricing behavior. However, excessively large price changes imply higher risks, which in turn incentivize sellers to slow down their adjustment speed in subsequent periods.

Accordingly, following standard formulations in the literature on bounded rationality and adaptive dynamic decision-making [3,4,8], we model the evolution of the adjustment speed as:

(12) $${\tilde{\omega }}_{i,t+1}=\max\left\{0,\left(1-\rho \right)\bar{\omega }+\rho \omega_{i,t}+\alpha \pi_{i,t}-\beta {\left(p_{i,t+1}-p_{i,t}\right)}^{2}\right\},i=1,2$$

Here, $\bar{\omega }>0$ denotes the baseline adjustment speed, ρ∈(0,1) captures inertia in adjustment speed, α>0 reflects the positive effect of profit on pricing aggressiveness, and β>0 measures the dampening effect of price fluctuations on adjustment speed. Since $p_{i,t+1}-p_{i,t}=0$ at the symmetric steady state, the volatility penalty term vanishes in equilibrium and therefore affects only off-equilibrium adjustment dynamics. The max operator ensures that adjustment speeds remain non-negative and economically feasible.

Since the platform imposes an upper bound Ω on the sellers’ price adjustment speeds, the actual adjustment speed adopted by the sellers is given by:

(13) $$\omega_{i,t+1}=\min\left\{\Omega ,{\tilde{\omega }}_{i,t+1}\right\},i=1,2$$

This rule implies that sellers may increase their adjustment speeds in response to market signals, but the actual speed cannot exceed the regulatory upper bound set by the platform. Combining the above equations, the overall dynamics of the system can be represented as:

(14) $$\left\{\begin{array}{l} p_{1,t+1}=p_{1,t}+\omega_{1,t}\frac{\partial \pi_{1,t}}{\partial p_{1,t}}\\ p_{2,t+1}=p_{2,t}+\omega_{2,t}\frac{\partial \pi_{2,t}}{\partial p_{2,t}}\\ \omega_{1,t+1}=\min\left\{\Omega ,\left(1-\rho \right)\bar{\omega }+\rho \omega_{1,t}+\alpha \pi_{1,t}-\beta {\left(p_{1,t+1}-p_{1,t}\right)}^{2}\right\}\\ \omega_{2,t+1}=\min\left\{\Omega ,\left(1-\rho \right)\bar{\omega }+\rho \omega_{2,t}+\alpha \pi_{2,t}-\beta {\left(p_{2,t+1}-p_{2,t}\right)}^{2}\right\} \end{array}\right.$$

Therefore, the system forms a four-dimensional discrete-time nonlinear dynamical system, with the state vector defined as $X_{t}=(p_{1,t},p_{2,t},\omega_{1,t},\omega_{2,t})$. Within this framework, the platform shapes sellers’ pricing behavior by selecting the regulatory upper bound, while the sellers’ endogenous adjustment speeds influence both market stability and profitability. This model provides the foundation for the subsequent equilibrium characterization, stability analysis, and the study of how platform regulation affects sellers’ profits and overall market dynamics.

## 4. Equilibrium and Stability Analysis

To analyze dynamic competition under platform regulation, we first consider the interior symmetric equilibrium when the platform-imposed upper bound on adjustment speed is non-binding near the steady state. Given symmetric seller parameters and identical platform rules, we focus on characterizing this symmetric equilibrium and subsequently examine its local stability.

### 4.1. Interior Symmetric Nash Equilibrium and Conditions for Local Stability

**Proposition** **1.** *If* 2b−d>0 *,* 0<ρ<1 *, and the resulting steady state satisfies* $q^{*}>0$ *and* $\omega^{*}>0$ *, then the system admits a unique interior symmetric equilibrium: *

$$E^{*}=(p^{*},p^{*},\omega^{*},\omega^{*})$$

*where* $p^{*}=\frac{(1-r)a+bc}{\left(1-r)(2b-d\right)}$ *,* $\omega^{*}=\bar{\omega }+\frac{\alpha }{1-\rho }\pi^{*}$ *,* $\pi^{*}=((1-r)p^{*}-c)(a-(b-d)p^{*})$ *.*

Proposition 1 shows that in a duopolistic market with substitutable products, sellers following a boundedly rational adjustment mechanism eventually converge to a symmetric long-run behavior. This convergence ensures that the model is well-defined from an economic perspective. From the equilibrium expression, the price $p^{*}$ is jointly determined by the underlying market structure parameters. In particular, (1−r) captures the compression effect of the platform’s commission on effective revenue, while the term 2b−d in the denominator reflects the “effective intensity” of market competition. As product substitutability (d) increases, 2b−d decreases, which in turn raises the equilibrium price level. This indicates that stronger substitution competition encourages sellers to raise prices to ease competitive pressure, creating an endogenous buffering mechanism. The proof is provided in Appendix A.1.

Meanwhile, the steady-state adjustment speed $\omega^{*}=\bar{\omega }+\frac{\alpha }{1-\rho }\pi^{*}$ clearly reveals the endogenous origin of pricing behavior: it is not only influenced by the baseline adjustment level, but is also closely related to profit levels and behavioral inertia. When profit $\pi^{*}$ is high or the inertia parameter ρ is large, the sellers’ adjustment speeds are significantly amplified.

This structure lays the foundation for the subsequent stability analysis, showing that the steady-state adjustment speed is determined endogenously by market profitability and behavioral parameters, rather than being exogenously imposed.

**Corollary** **1.** *Under *$\pi^{*}>0$*, we have *$\frac{\partial \omega^{*}}{\partial \bar{\omega }}>0$*,* $\frac{\partial \omega^{*}}{\partial \alpha }>0$*,* $\frac{\partial \omega^{*}}{\partial \rho }>0$*.*

Corollary 1 shows that, under $\pi^{*}>0$, the steady-state adjustment speed increases monotonically with the baseline adjustment level, profit sensitivity, and adjustment inertia. This implies that as long as the market remains profitable, sellers have an inherent incentive to choose higher steady-state adjustment speeds. The proof is provided in Appendix A.2.

From an economic perspective, this corollary characterizes an endogenous evolution path of “profit–acceleration–instability.” Higher profits enhance sellers’ confidence in pricing (through α), strengthen their reliance on past strategies (through ρ), and amplify the existing adjustment pace (through $\bar{\omega }$). The combined effect of these forces increases the endogenous steady-state adjustment speed, which may eventually push the system beyond the local stability boundary.

To examine stability at the equilibrium, we first derive the Jacobian matrix at the steady state:

(15) $$J(E^{*})=\left(\begin{array}{cccc} 1-2b(1-r)\omega^{*} & d(1-r)\omega^{*} & 0 & 0\\ d(1-r)\omega^{*} & 1-2b(1-r)\omega^{*} & 0 & 0\\ 0 & \alpha d((1-r)p^{*}-c) & \rho & 0\\ \alpha d((1-r)p^{*}-c) & 0 & 0 & \rho \end{array}\right)$$

Since $J(E^{*})$ is a block lower triangular matrix, its eigenvalues are determined by those of the diagonal blocks. The upper-left 2×2 price block has eigenvalues $\lambda_{1}=1-(2b-d)(1-r)\omega^{*}$ and $\lambda_{2}=1-(2b+d)(1-r)\omega^{*}$, while the lower-right 2×2 block has eigenvalues $\lambda_{3}=\rho$ and $\lambda_{4}=\rho$. Therefore, the stability of the equilibrium can be characterized by Proposition 2.

**Proposition** **2.** *Under *0<ρ<1*, the interior symmetric equilibrium *$E^{*}$is locally asymptotically stable if and only if $0<\omega^{*}<\frac{2}{\left(1-r)(2b+d\right)}$.

Proposition 2 characterizes the local stability condition conditional on a given steady-state adjustment speed $\omega^{*}$. The result shows that local stability depends critically on whether the endogenous steady-state adjustment speed remains within the stability region. Since $\omega^{*}=\bar{\omega }+\frac{\alpha }{1-\rho }\pi^{*}$, the steady-state adjustment speed is not exogenously fixed, but depends on profit incentives, adjustment inertia, and the baseline level of algorithmic aggressiveness. Combined with Corollary 1, this suggests that stronger profit incentives increase the endogenous steady-state adjustment speed. If this speed exceeds the local stability threshold, the system may become unstable. The proposition therefore provides an analytical characterization of the local stability condition for a given steady-state adjustment speed. The proof is provided in Appendix A.3.

**Figure 2:**
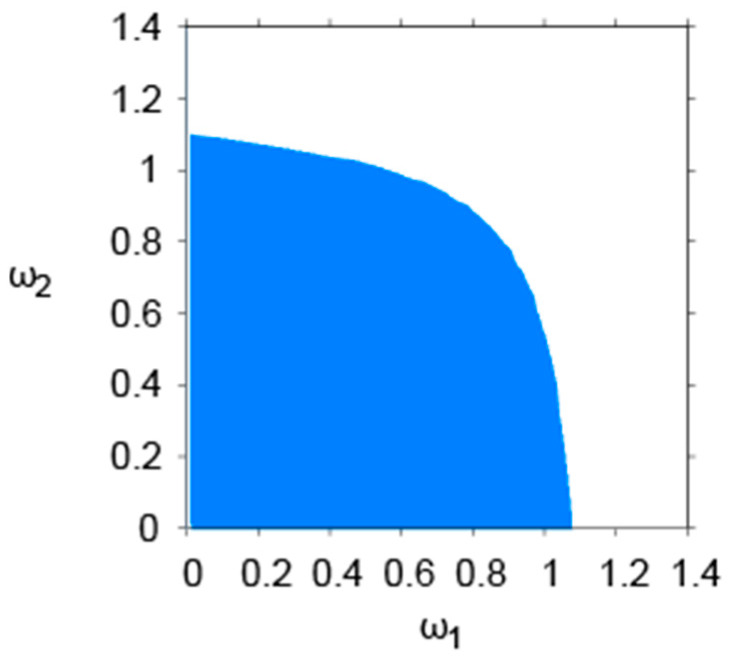
Stability Region of the System in the $\left(\omega_{1},\omega_{2}\right)$ Space (a=10, b=1, d=0.55, c=2, r=0.10, ρ=0.45, $\bar{\omega }=0.03$, α=0.01, β=0.04, Ω=10, $p_{1}=8.0$, $p_{2}=8.2$).

To present these theoretical results more intuitively and to illustrate the joint impact of the two sellers’ adjustment speeds on system stability, Figure 2 further depicts the stability region in the $\left(\omega_{1},\omega_{2}\right)$ space. It can be observed that the stability region exhibits a distinctly nonlinear boundary. As the adjustment speeds $\omega_{1}$ and $\omega_{2}$ increase, the system gradually transitions from a stable state to an unstable one. This indicates that excessively high adjustment speeds tend to undermine system stability, whereas relatively low adjustment speeds help maintain a stable market environment. Moreover, the asymmetry of the stability region reflects the interaction between the two sellers’ pricing behaviors, suggesting that a change in one seller’s adjustment speed can affect the overall system stability through competitive dynamics.

### 4.2. Platform Regulation and Stability Restoration

Next, we consider the role of the platform’s speed cap Ω. When the platform imposes regulation, the sellers’ actual adjustment speed becomes $\omega_{i,t+1}=\min\left\{\Omega ,\left(1-p\right)\bar{\omega }+p\omega_{i,t}+\alpha \pi_{i,t}-\beta {\left(p_{i,t+1}-p_{i,t}\right)}^{2}\right\},i=1,2$. If, in the absence of regulation, the sellers’ endogenous steady-state adjustment speed already exceeds the stability threshold, the platform can restore local stability by imposing a suitably chosen binding upper bound that truncates the actual adjustment speed to within the stable region. Let $\omega^{u}=\bar{\omega }+\frac{\alpha }{1-\rho }\pi^{*}$ denote the interior steady-state adjustment speed in the absence of regulation.

**Proposition** **3.** *If in the absence of regulation *$\omega^{u}>\frac{2}{\left(1-r)(2b+d\right)}$*, then there exists an effective binding upper bound *Ω *that can restore local asymptotic stability of the system. More specifically, as long as the platform is set as follows: *

$$0<\Omega <\frac{2}{\left(1-r)(2b+d\right)}$$

*then this upper bound is binding in the neighborhood of the steady state, and the system regains local stability.*

Proposition 3 shows that instability may emerge when the endogenous steady-state adjustment speed becomes excessively large relative to the local stability region. Since stronger profit incentives tend to increase adjustment aggressiveness, the equilibrium may eventually fall outside the stability region in the absence of regulation. By imposing an upper bound on adjustment speeds, the platform can effectively restrict the realized adjustment intensity within the stable region and thereby restore local stability. The proof is provided in Appendix A.4.

In the preceding analysis, the platform influences sellers’ dynamic pricing behavior and system stability by setting an upper bound Ω on price adjustment speeds. Since regulation can both curb excessive acceleration and reduce market volatility, while at the same time weakening normal price responsiveness and incurring additional regulatory costs, the platform’s profit-maximizing regulatory intensity does not necessarily coincide with the socially optimal level of regulation.

To this end, we define the platform’s long-run average profit $\bar{\Pi }\left(\Omega \right)$ under a given Ω as:

(16) $$\bar{\Pi }\left(\Omega \right)=\underset{T\to \infty }{\lim}\sup\frac{1}{T}\sum_{t=1}^{T}\left[r\left(p_{1,t}q_{1,t}+p_{2,t}q_{2,t}\right)-C\left(\Omega \right)\right]$$

Accordingly, the platform’s optimal regulatory intensity $\Omega^{P}$ is defined as:

(17) $$\Omega^{P}=\arg\underset{\Omega \in \left[0,\Omega^{\max}\right]}{\max}\bar{\Pi }\left(\Omega \right)$$

Similarly, we define the system’s long-run average social welfare $\bar{W}\left(\Omega \right)$ under a given Ω, as well as the socially optimal regulatory intensity $\Omega^{S}$, as:

(18) $$\bar{W}\left(\Omega \right)=\underset{T\to \infty }{\lim\sup}\frac{1}{T}\sum_{t=1}^{T}W_{t}\left(\Omega \right)$$

(19) $$\Omega^{S}=\arg\underset{\Omega \in \left[0,\Omega^{\max}\right]}{\max}\bar{W}\left(\Omega \right)$$

among them, $W_{t}(\Omega )=\left(p_{1,t}-c\right)q_{1,t}+\left(p_{2,t}-c\right)q_{2,t}+CS_{t}-C\left(\Omega \right)$. It is worth noting that the platform’s commission revenue essentially constitutes a transfer payment between the platform and the sellers, and therefore should not be double-counted in the calculation of social welfare.

Equations (16) and (18) can be rewritten as:

$$\bar{\Pi }\left(\Omega \right)=r\bar{R}\left(\Omega \right)-C\left(\Omega \right)$$

$$\bar{W}\left(\Omega \right)=\bar{PS}\left(\Omega \right)+\bar{CS}\left(\Omega \right)-C\left(\Omega \right)$$

Section 5 uses numerical simulations to examine how seller profit-driven endogenous acceleration moves the system from stability to cyclical oscillations and complex dynamics. It also analyzes the effects of platform regulation on seller profits, platform profits, and social welfare. Because the introduction of nonlinear adjustment dynamics, entropy-triggered control, and volatility-related welfare costs makes analytical characterization of socially and platform-optimal regulation levels highly intractable, numerical simulations are used to evaluate governance outcomes under different regulation intensities.

## 5. Numerical Simulation Analysis

To verify the dynamic properties of the model and the results of the theoretical analysis, this paper conducts numerical simulations based on a set of representative parameters. The baseline parameter values are selected with three considerations. First, they satisfy the standard regularity conditions of a differentiated duopoly market [34], including positive demand, positive seller profits, and the existence of an interior equilibrium. Second, they describe an economically meaningful platform market with moderate product substitutability and a non-extreme commission rate. Third, they allow the system to exhibit representative dynamic transitions from local stability to bifurcation and chaos, which are commonly examined in the bounded-rationality and complex-dynamics literature [3,4,7].

Specifically, in the market demand function, the baseline market size is set to a=10, and the own-price sensitivity coefficient is set to b=1. These values normalize the demand scale and ensure that demand decreases at a moderate rate as sellers raise prices. The cross-price influence coefficient is set to d=0.55, which captures a moderate degree of product substitutability while satisfying the standard condition 0<d<b. The unit production cost is set to c=2 to ensure positive operating margins around the interior equilibrium. The platform commission rate is set to r=0.10, representing a moderate commission level that does not dominate sellers’ pricing incentives.

Regarding the adjustment-speed dynamics, the speed inertia parameter is set to ρ=0.45, indicating that sellers partially rely on past adjustment behavior. The benchmark adjustment speed is set to $\bar{\omega }=0.03$, representing a relatively conservative pricing adjustment tendency in the absence of strong profit incentives. The profit-driven coefficient α serves as the key bifurcation parameter and varies within the interval [0,0.025] to characterize different levels of sellers’ sensitivity to profit signals. The price-fluctuation penalty coefficient is set to β=0.04 to reflect the adjustment risk associated with excessive price fluctuations. Under the baseline setting, the theoretical regulatory upper bound is fixed at $\Omega^{\max}=10$. In the numerical experiments where regulation intensity is treated as a bifurcation parameter, $\Omega^{\max}$ varies over the interval [0,1.2] to examine how different levels of platform regulation affect market stability. The selected parameter ranges guarantee numerical convergence and avoid economically meaningless outcomes such as negative demand or explosive divergence. The parameter configuration is not intended to match a specific empirical platform market, but rather to provide a stylized environment for illustrating the underlying dynamic mechanisms.

The initial states are set as $p_{1}=8.0$ and $p_{2}=8.2$, and the initial values of the price adjustment rates are $\omega_{1}=0.03$ and $\omega_{2}=0.035$, respectively. To eliminate the influence of the initial conditions on the results, a certain number of pre-iterations (burn-in) are first performed during the simulation, followed by recording the long-term dynamic behavior of the system.

### 5.1. Role of Pricing Aggressiveness

**Figure 3:**
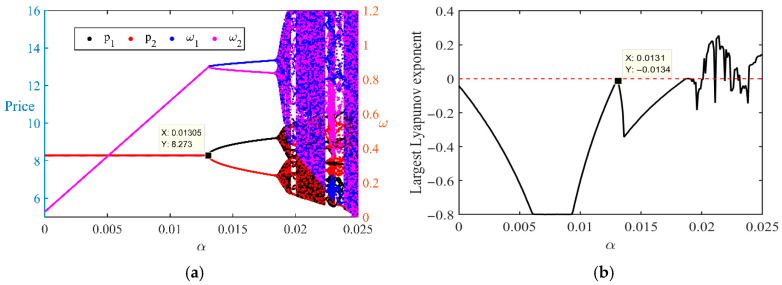
(**a**) Bifurcation Diagram and (**b**) largest Lyapunov Exponent with Respect to Pricing Aggressiveness α.

To examine the impact of profit-driven price aggressiveness on the dynamic evolution of the system, this paper uses parameter α (pricing aggressiveness) as the bifurcation parameter to plot the price path and the maximum Lyapunov exponent, as shown in Figure 3.

Figure 3a illustrates the evolution of price trajectories for seller 1 ($p_{1}$) and seller 2 ($p_{2}$) under different values of α, which captures the positive effect of profit on pricing aggressiveness. As α increases, the price paths gradually exhibit stronger fluctuations, and for higher values of α clear nonlinear patterns begin to emerge. In particular, when α approaches 0.013, the price trajectories display pronounced oscillations, followed by a relatively stable pattern thereafter. This indicates that as pricing aggressiveness (α) increases, price volatility increases, market competition intensifies, and market instability increases. The non-linear market fluctuations may be due to competitive pressure caused by excessively high pricing aggressiveness, leading to sharp price fluctuations in the short term. This phenomenon clearly demonstrates the positive impact of α on market pricing behavior; a higher α value means that sellers are more aggressive in pricing, thereby exacerbating market price volatility.

Figure 3b shows the variation of the Lyapunov exponent with respect to α. The Lyapunov exponent measures the stability of the system; a larger Lyapunov exponent indicates that the system is more likely to enter a chaotic state. As shown in Figure 3b, when α approaches 0.013, the Lyapunov exponent increases significantly and exhibits pronounced oscillatory patterns around this value, indicating a substantial decline in system stability. This phenomenon suggests that when α is relatively high, the dynamic stability of the market system weakens and the system enters a chaotic state, with a substantial increase in the uncertainty of price fluctuations. The increase in α directly leads to a deterioration in system stability, making market behavior more unpredictable. This indicates that when pricing aggressiveness is excessively high, the market no longer maintains sufficient stability and is prone to falling into disorder and chaos.

In this context, pricing aggressiveness (α) not only affects the volatility of price trajectories but also influences overall market stability. Higher values of α lead to greater price volatility and increased market uncertainty, which may pose significant risks for both platforms and sellers. Therefore, platforms should pay close attention to the control of pricing aggressiveness. In particular, when α is excessively high, appropriate regulatory measures should be considered to prevent the market from falling into a chaotic and unstable state.

From an economic perspective, entropy measures the degree of uncertainty and unpredictability in market pricing dynamics. When the system remains stable, price trajectories are relatively regular and predictable, corresponding to lower entropy levels and stronger market coordination. In contrast, as profit-driven adjustment speeds push the system toward bifurcation or chaos, pricing trajectories become increasingly irregular and difficult to anticipate, reflecting the expansion of dynamic uncertainty in the market.

Different entropy measures capture different aspects of market complexity. Shannon entropy reflects the uncertainty of price distributions, permutation entropy captures the temporal irregularity of pricing trajectories, and joint entropy measures the overall coordination complexity across sellers. Therefore, entropy complements traditional stability indicators such as bifurcation diagrams and Lyapunov exponents by providing an economically meaningful perspective on market predictability, coordination difficulty, and platform governance effectiveness.

**Figure 4:**
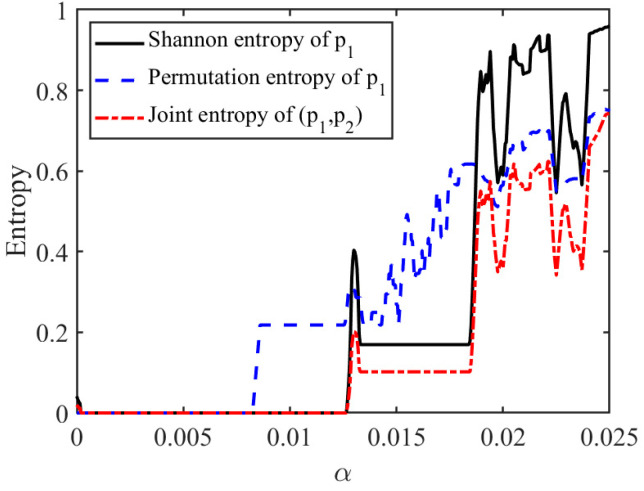
Entropy measures of the price dynamics with respect to pricing aggressiveness α.

Figure 4 illustrates the evolution of Shannon entropy, permutation entropy, and joint entropy as the profit-driven parameter changes. It can be seen that in the lower α range, all three entropy values are close to 0, indicating that the system’s orbits are highly concentrated, price dynamics are relatively stable, and the system is in a low-complexity state. As α increases, the three entropy indices begin to rise significantly and remain at a high level in the higher range, indicating that the system gradually evolves from stable equilibrium to a periodic oscillation or even a chaotic state. The price orbits are more dispersed in the state space, and dynamic uncertainty is significantly enhanced.

Across the different entropy measures, permutation entropy begins to rise relatively earlier, indicating that it is more sensitive to changes in the temporal structural complexity of the price series. By contrast, Shannon entropy and joint entropy more directly capture the degree of dispersion in the system’s state distribution. Although the three entropy measures differ in their numerical levels, they exhibit a broadly consistent overall trend, suggesting that profit-driven pricing aggressiveness continuously increases system complexity.

The simultaneous increase in Shannon entropy, permutation entropy, and joint entropy suggests that the market becomes not only more volatile, but also more difficult to predict and coordinate. In particular, the rise in permutation entropy indicates that pricing trajectories gradually lose temporal regularity, while the increase in joint entropy reflects growing coordination complexity across competing sellers. Economically, this implies that excessive profit-driven acceleration weakens market predictability and increases the difficulty of platform governance. Therefore, entropy serves as a useful complement to bifurcation diagrams and the largest Lyapunov exponent by capturing the informational complexity embedded in platform pricing dynamics. It also provides a basis for the subsequent analysis of how platform regulation and feedback control mechanisms can suppress instability and reduce dynamic uncertainty.

**Figure 5:**
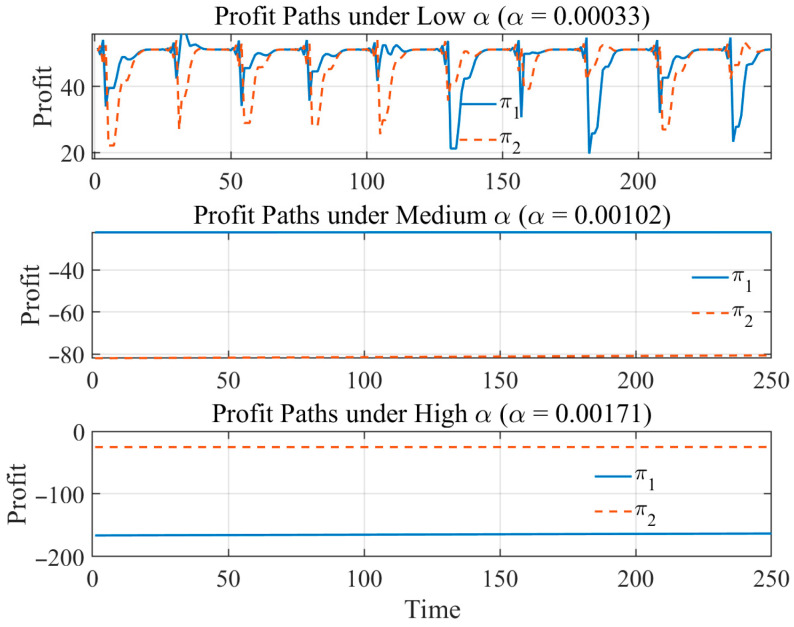
Profit Dynamics under Different Levels of Pricing Aggressiveness α.

Figure 5 illustrates the dynamic evolution paths of the profits of the two sellers under different levels of pricing aggressiveness α, illustrating how higher pricing aggressiveness gradually pushes the market from a relatively stable profit regime toward a low-profit regime.

When α is at a relatively low level, profits exhibit regular periodic fluctuations with limited amplitude and a clear repetitive structure. This indicates that under a relatively mild adjustment mechanism, price updates remain sufficiently moderate, and the system operates within a stable bounded fluctuation regime. At this stage, profit-driven adjustment plays a “fine-tuning” role that helps maintain market coordination and relatively stable profitability. When α increases to a moderate level, the profit trajectories rapidly converge to a lower level and lose their original periodic structure. This suggests that excessively strong profit incentives induce over-adjustment in prices, causing sellers to deviate persistently from the high-profit region and thereby intensifying competition and compressing profit margins. In this stage, the role of profit signals shifts from facilitating gradual coordination to generating excessive pricing responses, resulting in deterioration of market performance. As α increases further, the system enters an extremely low-profit regime and remains trapped at a persistently low level over the long run. Profits no longer exhibit noticeable fluctuations but instead converge to a low-profit state, indicating that excessive pricing aggressiveness may lead the market into a self-reinforcing pattern of over-adjustment and profit deterioration. At this stage, price adjustment no longer improves market coordination and instead prevents the market from returning to a high-profit operating region.

Therefore, pricing aggressiveness is not necessarily beneficial when excessively large; its effect on profits exhibits a clear nonlinear pattern. Relatively low levels of α help maintain stable market responsiveness, whereas excessively high α weakens profitability and pushes the system toward a persistently low-profit regime. These results suggest that profit-driven adjustment can play both a coordination-enhancing role and a profit-deterioration role, implying a trade-off between market responsiveness and long-run profitability.

### 5.2. Impact of Platform Regulation

**Figure 6:**
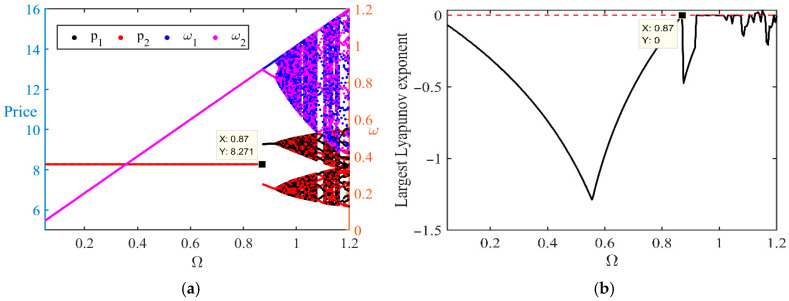
(**a**) Bifurcation Diagram and (**b**) largest Lyapunov Exponent with Respect to Regulation Intensity Ω.

Figure 6 illustrates the relationship between price trajectories and the Lyapunov exponent, analyzing the impact of different values of Ω (the platform control parameter) on market dynamics. Figure 6a presents the price trajectories of seller 1 ($p_{1}$) and seller 2 ($p_{2}$) under varying values of Ω It can be observed that as Ω increases, fluctuations in the price trajectories become more pronounced. In particular, when Ω approaches 0.87, the price paths exhibit clear nonlinear fluctuations. This indicates that as platform intervention in the market gradually weakens (i.e., when Ω is relatively high), market pricing becomes more sensitive and volatile, leading to sharp price fluctuations and increased uncertainty. Figure 6b shows the variation of the Lyapunov exponent with respect to Ω. The Lyapunov exponent serves as a measure of system stability: as its value increases, the system becomes more likely to enter a chaotic state. From the figure, it can be seen that when Ω approaches 0.87, the Lyapunov exponent rises sharply, indicating a significant decline in system stability and the onset of chaos. Changes in Ω therefore have a substantial impact on market stability. Higher values of Ω imply weaker platform regulation, under which the market exhibits stronger nonlinear fluctuations and greater unpredictability, increasing risk for both sellers and the platform. In contrast, lower values of Ω indicate stronger platform control, under which price and profit trajectories are more stable and overall market stability is higher.

Therefore, the platform should exercise caution in selecting Ω to avoid excessive deregulation that may lead to market instability. Overall, an appropriate choice of the platform control parameter Ω is crucial for maintaining market stability and predictability. In particular, when the market experiences large fluctuations, moderate regulation may be necessary to ensure system stability.

**Figure 7:**
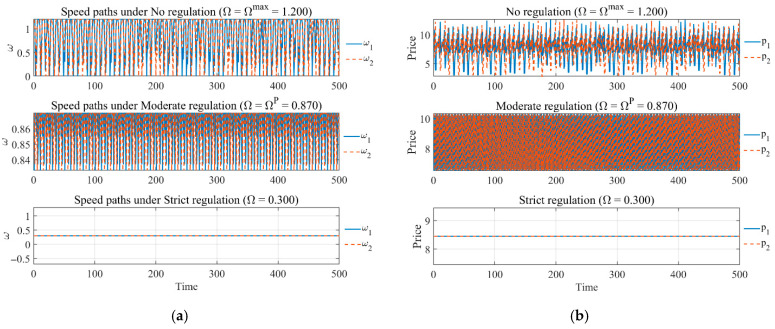
(**a**) Adjustment-speed dynamics and (**b**) price dynamics under no regulation, moderate regulation, and strict regulation.

Figure 7 presents the price trajectories of sellers under three different regulatory scenarios: no regulation, moderate regulation, and strict regulation. In the absence of regulation, the value of Ω is at its maximum (i.e., Ω = $\Omega^{\max}$= 1.200). In this case, the price paths exhibit high-frequency fluctuations and pronounced periodic variations, indicating that market prices are easily affected by excessive competition in the absence of regulation, leading to large fluctuations. As regulation is gradually strengthened (Ω = $\Omega^{\max}$ = 0.870), price fluctuations decrease and tend to stabilize, and the price paths become smoother. This suggests that moderate regulation can effectively mitigate drastic price fluctuations and help maintain a certain level of market stability. Under strict regulation (Ω = 0.300), prices converge to a stable level and exhibit almost no variation, reflecting a high degree of price stability. For the market, although excessive regulation can suppress price fluctuations, it may also lead to price rigidity and reduce the flexibility of market responses.

**Figure 8:**
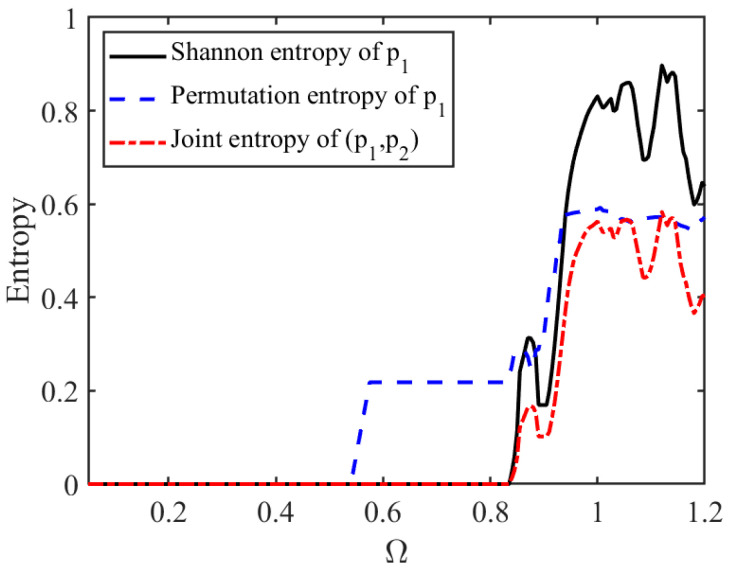
Entropy measures of the system under different platform regulation levels Ω.

Figure 8 illustrates the evolution of three types of entropy measures with respect to the platform regulation upper bound Ω. It can be observed that within the lower range of Ω, Shannon entropy, permutation entropy, and joint entropy all remain at relatively low levels, indicating that under strict regulation, the system dynamics are relatively stable, price trajectories are concentrated, and overall complexity is low. As Ω gradually increases, all three entropy measures rise significantly and exhibit substantial fluctuations in the higher range, suggesting that as regulation is relaxed, system trajectories gradually disperse in the state space and dynamic uncertainty increases markedly. The consistent trends across the three entropy measures further indicate that platform regulation intensity has a significant impact on system complexity.

These results suggest that constraints on adjustment speed imposed by the platform not only stabilize the system but also reduce market complexity from an informational perspective. By limiting excessive acceleration in price adjustment, strong regulation effectively suppresses the disordered expansion of price dynamics and maintains the system at relatively low levels of Shannon entropy, permutation entropy, and joint entropy. This indicates that strong regulation not only improves the predictability of market price outcomes, but also enhances the temporal regularity of price evolution and reduces the coordination complexity among competing sellers, thereby mitigating dynamic uncertainty in the market. In contrast, relaxed regulation may lead to dispersed price dynamics, irregular pricing behavior, and greater coordination difficulty among market participants. Therefore, from the perspective of complexity governance, platforms need to strike a balance between flexibility and stability, avoiding excessive deregulation that may induce systemic instability.

**Figure 9:**
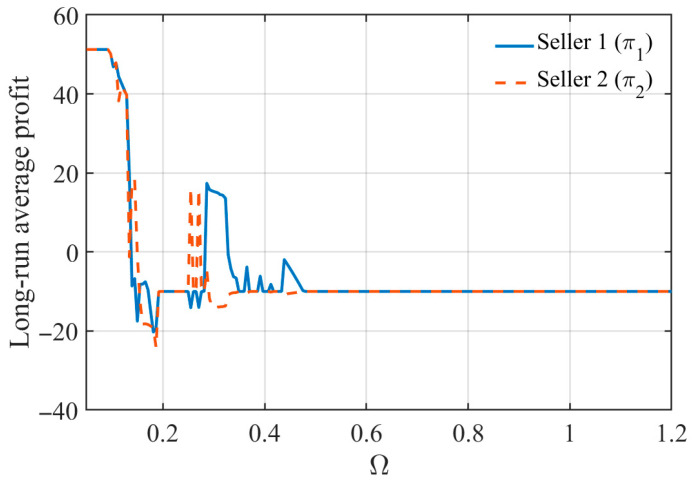
Long-Run Average Profit under Different Regulation Levels Ω.

Figure 9 illustrates the relationship between seller profits and the platform-imposed regulation intensity Ω. The blue solid line and the red dashed line represent the long-run average profits of Seller 1 and Seller 2, respectively. The results indicate that regulation intensity has a substantial impact on profit dynamics and market outcomes.

When Ω is relatively small, the platform imposes a strict upper bound on adjustment speeds, which effectively restrains excessive pricing responses and helps maintain relatively stable and high profit levels. As Ω increases, the regulatory constraint gradually weakens, and seller profits begin to exhibit noticeable fluctuations and deterioration. In the intermediate region, excessive adjustment flexibility leads to intensified competition and unstable profit performance. When Ω becomes sufficiently large, the system eventually converges to a persistently low-profit regime.

These results suggest that weakening regulation does not necessarily improve long-run market performance. Instead, excessive pricing flexibility may induce over-adjustment behavior and persistent profit deterioration. Therefore, platform regulation primarily plays a stabilizing role by preventing adjustment speeds from becoming excessively aggressive and by mitigating long-run profit collapse.

**Figure 10:**
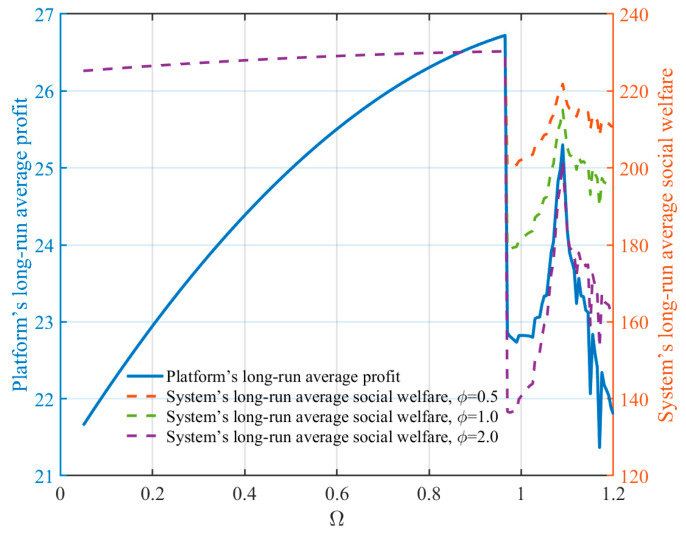
Platform objective and social welfare under different regulation intensities.

Figure 10 compares the platform objective and the volatility-adjusted social welfare objective under different regulation intensities. Since the welfare objective additionally incorporates producer surplus, consumer surplus, and volatility-related welfare losses, its numerical magnitude is naturally larger than that of the platform objective. Therefore, the two objectives are reported using separate vertical axes for visual comparison.

As the regulation intensity varies, both the platform objective and the welfare objective exhibit noticeable changes near the instability region. In particular, when regulation becomes excessively weak and the system approaches unstable pricing dynamics, both objectives decline due to intensified price fluctuations. Moreover, higher values of the volatility-cost parameter ϕ make the welfare objective more sensitive to market unpredictability.

These results suggest that excessive pricing volatility not only reduces platform profitability, but also generates welfare losses by weakening market predictability and increasing coordination difficulty among market participants. Therefore, appropriate regulation can help stabilize the pricing system and mitigate volatility, whereas overly weak regulation may push the market into a high-volatility and low-welfare regime.

### 5.3. Chaos Control

In the previous analysis, we have shown that when the profit-driven parameter or the adjustment speed exceeds a critical threshold, the system undergoes transitions from stability to periodic bifurcation and eventually to chaos. A natural question therefore arises: when the entire market falls into chaos, does the platform have effective means to control it?

Following the permutation-entropy approach of Bandt and Pompe [44], we use (PE(⋅)) to characterize the temporal irregularity of recent pricing trajectories. Let $H_{t}$ the normalized permutation-entropy indicator of recent pricing trajectories at time t, defined as $H_{t}=\frac{1}{2}[PE(p_{1,t-L+1:t})+PE(p_{2,t-L+1:t})]$, where the entropy is computed over a rolling observation window of length L. The platform specifies a tolerance threshold $\bar{H}$ for pricing irregularity. When $H_{t}\leq \bar{H}$, the pricing process is regarded as sufficiently regular and predictable, and no additional intervention is activated. When $H_{t}>\bar{H}$ indicating excessive irregularity in price evolution, the platform activates the speed-damping control mechanism to suppress further instability.

To mitigate profit-driven excessive acceleration, we adopt a feedback control approach from the economic chaos literature. A negative feedback term, based on the deviation of adjustment speed from a target level, is added to the adjustment-speed evolution equation. Combined with the upper bound on adjustment speed imposed by the platform, we construct a damping control mechanism based on endogenous adjustment speed [45]. The basic idea is as follows: since the direct source of system instability is not the price variable itself, but rather the continuous increase in adjustment speed driven by profit incentives, which amplifies price jumps and competitive fluctuations, the key to chaos control does not lie in directly correcting price levels, but in imposing appropriate damping on endogenous adjustment speed so as to bring it back to the stable region. Based on this, we assume that in each period of speed updating, the platform or the sellers’ algorithm introduces a negative feedback control term according to the deviation between the current adjustment speed and the target speed, so as to suppress excessive acceleration behavior. Specifically, on the basis of the original updating rule, the evolution equation of the adjustment speed for seller i is modified as follows:

(20) $$\omega_{i,t+1}=\max\left\{0,\min\left\{\Omega ,\left(1-\rho \right)\bar{\omega }+\rho \omega_{i,t}+\alpha \pi_{i,t}-\beta {\left(p_{i,t+1}-p_{i,t}\right)}^{2}-\theta_{t}(\omega_{i,t}-\omega^{b})\right\}\right\},i=1,2$$

where $\theta_{t}=\theta \max\left\{\frac{H_{t}-\bar{H}}{1-\bar{H}},0\right\}$.θ denotes the maximum feedback-control intensity, whereas the effective control strength $\theta_{t}$ is endogenously activated according to the entropy-trigger condition. $\omega^{b}$ denotes the target adjustment speed. From an economic perspective, the control term adjusts the system as follows. If the actual adjustment speed exceeds the target, the negative feedback reduces it in the next period. If the speed falls below the target, the feedback is relaxed, allowing normal market responses to proceed without excessive damping.

In the entropy-triggered control simulations, the rolling observation window length is set to (L=80), which balances the identification of temporal irregularity and the responsiveness of the control mechanism. The entropy threshold is set to ($\bar{H}=0.45$). Since the normalized permutation entropy lies within the interval [0,1], this moderate threshold allows a certain degree of pricing flexibility while activating intervention once pricing trajectories become sufficiently irregular and difficult to predict.

**Figure 11:**
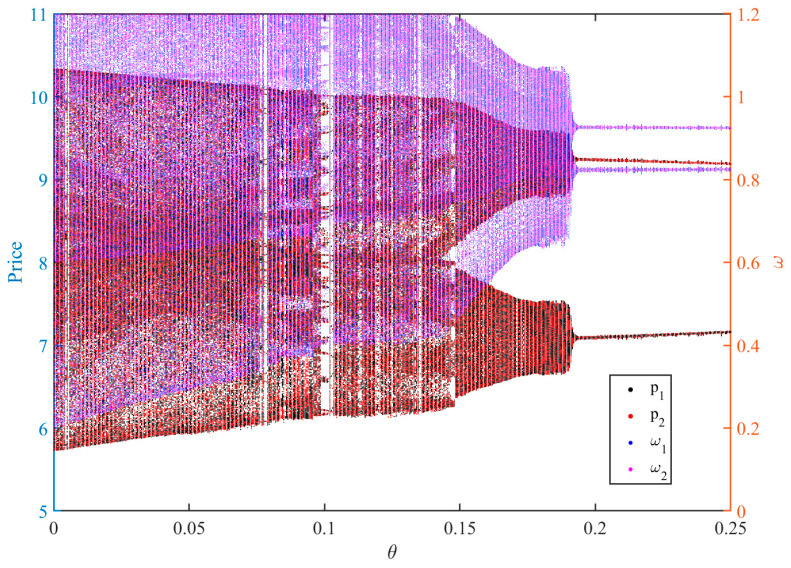
Bifurcation diagram under different maximum entropy-triggered feedback-control intensities θ.

Figure 11 simultaneously depicts the chaotic control diagrams of the price variables $p_{i}$ and the adjustment speed variables $\omega_{i}$ with respect to the control strength θ. When θ is small, the left-hand side of the figure exhibits a highly dense and irregular cloud of points. Both the price and adjustment speed variables display evident non-periodic fluctuations, indicating that the system is in a typical chaotic state. At this stage, driven by profit incentives, sellers continuously increase their adjustment speeds, resulting in excessive jumps in price updates, and the market competition exhibits strong instability and unpredictability. As θ gradually increases, the point cloud begins to contract and a clear layered structure emerges. The system undergoes an evolutionary path from high-period to low-period dynamics, suggesting that the control mechanism gradually weakens the original nonlinear amplification effect. With a further increase in θ, both prices and adjustment speeds eventually converge to a single stable point, and the system restores local asymptotic stability.

Therefore, from the perspective of platform pricing-system design, platforms should treat “price-adjustment-speed control” as a core governance tool, rather than focusing solely on price levels themselves. Specifically, platforms can introduce an entropy-triggered adaptive regulation mechanism similar to that proposed in this paper at the algorithmic level. By continuously monitoring the temporal irregularity and unpredictability of pricing trajectories through entropy indicators, the platform can identify early signals of excessive market instability. Once the entropy level exceeds the platform’s tolerance threshold, the system can automatically impose damping on sellers’ adjustment speeds through algorithmic rules, thereby suppressing excessively aggressive pricing behavior and mitigating sharp short-term price fluctuations.

Such a mechanism represents a form of “soft regulation.” Rather than directly restricting sellers’ pricing freedom, it adaptively intervenes only when pricing dynamics become excessively irregular, allowing the market to retain a certain degree of flexibility while maintaining operation near the stable region. This result suggests that, in digital platform markets, regulating the dynamics and complexity of price adjustment may be more effective than directly constraining price levels themselves.

### 5.4. Robustness Checks

To examine whether the main findings depend on the baseline parameter configuration, this paper further conducts robustness checks by varying two key parameters: the substitution intensity d and the platform commission rate r. These two parameters are particularly important because d determines the strength of market competition, while r affects the distribution of transaction revenue between sellers and the platform.

**Figure 12:**
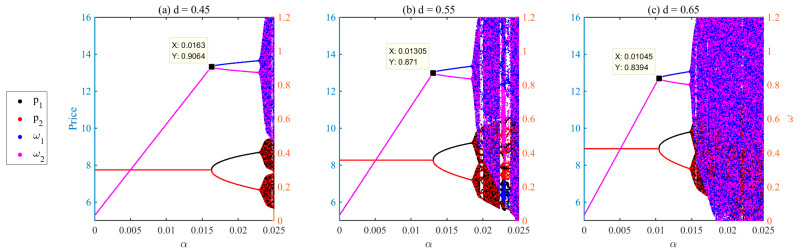
Robustness check under different product substitutability levels d.

First, the substitution parameter is varied from the baseline value d=0.55 to d=0.45 and d=0.65. Figure 12 shows that increasing substitutability lowers the bifurcation threshold: instability emerges around α≈0.0163 for d=0.45, α≈0.01305 for d=0.55, and α≈0.01045 for d=0.65. The qualitative dynamic mechanism remains unchanged. Higher substitutability strengthens competitive interaction, making the system more sensitive to profit-driven acceleration, whereas lower substitutability enlarges the stable region and delays bifurcations. Despite shifts in exact thresholds, the main conclusion holds: excessive endogenous adjustment speed amplifies price fluctuations and can destabilize the market.

**Figure 13:**
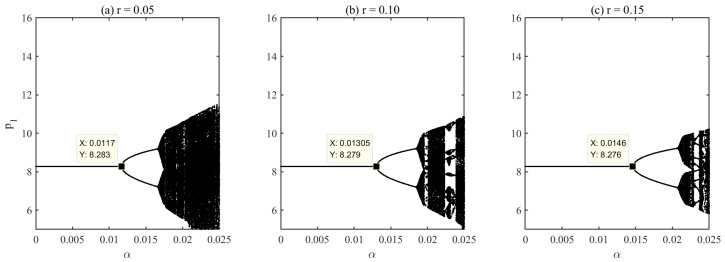
Robustness checks under different platform commission rates r.

Second, the platform commission rate is varied from the baseline value r=0.10 to r=0.05 and r=0.15. Figure 13 presents the robustness results under these different rates. The results show that the commission rate shifts the precise bifurcation threshold, while the qualitative dynamic mechanism remains unchanged. Specifically, the bifurcation occurs at α≈0.0117 when r=0.05, at α≈0.01305 when r=0.10, and at α≈0.0146 when r=0.15. Lower commission rates allow sellers to retain more revenue, strengthening profit-driven adjustment incentives and causing instability to emerge at lower pricing aggressiveness. In contrast, higher commission rates reduce effective incentives and delay the onset of instability. Despite changes in the exact bifurcation points, the main conclusions remain robust: endogenous adjustment speed continues to drive instability, and platform-imposed speed regulation effectively suppresses excessive fluctuations and restores system stability across different commission settings.

Although the precise bifurcation points vary with parameters, the main conclusions on endogenous acceleration, and market stability remain qualitatively robust.

## 6. Conclusions

This paper studies dynamic pricing in platform markets by developing a discrete-time model that integrates a Stackelberg game with bounded rational adjustment mechanisms. From a unified perspective of endogenous adjustment speed, platform regulation, and chaos control, we systematically examine the relationship between market stability and platform governance.

Our results show that adjustment speed is a key determinant of system stability. Even if the market starts in a stable region, profit-driven incentives can cause sellers to increase pricing aggressiveness. This may push the system beyond the stability threshold, resulting in periodic oscillations or chaotic dynamics and higher system complexity. This finding provides a behavioral explanation for the emergence of complex dynamics and extends the existing literature, which primarily attributes instability to exogenous parameter changes.

We further show that platform regulation can serve as an effective tool for restoring stability. By imposing an upper bound on adjustment speed, the platform can create a binding constraint near the steady state. This keeps the system within the stable region, reduces entropy, and limits dynamic uncertainty. However, regulation is not monotonically beneficial: moderate intervention helps balance stability and market responsiveness, whereas excessive regulation may dampen normal price adjustment mechanisms and reduce market efficiency.

In addition, our numerical analyses show that excessive pricing volatility can generate substantial welfare losses by reducing market predictability and increasing coordination difficulty among market participants. While the platform primarily determines its regulatory intensity based on profit considerations, social welfare additionally accounts for stability and volatility-related costs. This highlights the importance of designing regulatory mechanisms that balance market stability, pricing flexibility, and welfare objectives under dynamic conditions.

Building on these insights, we introduce an entropy-triggered adaptive feedback-control mechanism that stabilizes the system by monitoring the irregularity of pricing trajectories and activating damping only when excessive volatility is detected. This approach mitigates chaotic dynamics, preserves pricing flexibility, and reduces both entropy and market complexity. From a policy perspective, controlling the dynamics and complexity of price adjustments may be more effective for maintaining stable and efficient platform markets than imposing static price constraints.

From a policy and managerial perspective, the results suggest several implications for the governance of algorithmic pricing systems. First, platforms should not focus solely on static price levels, but also monitor the dynamics of price adjustment itself. Excessively aggressive adjustment speeds may amplify market instability and generate chaotic fluctuations, even when the underlying market environment remains unchanged. Second, rather than imposing rigid price controls, platforms may achieve better outcomes through adaptive regulation mechanisms that directly constrain excessive adjustment-speed acceleration while preserving normal market responsiveness. Third, entropy-based indicators can serve as early-warning signals of rising market complexity and instability, enabling platforms to implement intervention only when pricing dynamics become sufficiently irregular. Finally, because platform incentives may not fully internalize the welfare losses associated with excessive volatility and market instability, external regulatory oversight may still be necessary in highly algorithmic pricing environments.

The robustness analyses further demonstrate that these conclusions remain qualitatively consistent under alternative levels of product substitutability and platform commission rates, suggesting that the identified instability mechanism is not sensitive to a particular baseline parameter setting.

This study also has several limitations. First, the model focuses on a symmetric duopoly setting to preserve analytical tractability and to clearly characterize the interaction between endogenous adjustment speed, platform regulation, and system stability. In a multi-seller platform environment, the dimensionality of the dynamic system would increase substantially, and heterogeneous interactions among sellers may generate richer nonlinear dynamics, including asymmetric bifurcations, clustering behavior, and network spillover effects. Second, the current framework is primarily theoretical and simulation-based, without empirical calibration using real platform pricing data. In addition, although the model incorporates bounded rational adaptive pricing behavior, it does not explicitly consider forward-looking learning mechanisms or stochastic demand shocks. Future research may extend the framework by incorporating seller heterogeneity, learning dynamics, stochastic market environments, and empirical validation using real-world platform data to enhance external validity and practical applicability. In particular, a promising direction is to further investigate how different welfare structures, volatility externalities, and regulatory objectives influence the divergence between privately optimal and socially desirable platform governance policies in more complex market environments.

## Figures and Tables

**Table 1:** Summary of notations.

Symbol	Descriptions
i=1,2	Index of sellers
t	Discrete time period
$p_{i,t}$	Price of seller i at time t
$q_{i,t}$	Demand faced by seller i
r	Commission rate
a	Base market demand level
b	Own-price sensitivity coefficient
d	Cross-price effect (degree of substitutability)
c	Unit production cost
U	Utility function
m	Residual income (numeraire good)
$\pi_{i,t}$	Profit of seller i at time t
$\Pi_{t}$	Platform profit at time t
$CS_{t}$	Consumer surplus at time t
$W_{t}$	Social welfare at time t
$\bar{\omega }$	Baseline adjustment speed
$\omega_{i,t}$	Price adjustment speed of seller i at time t
α	Profit-driven sensitivity of adjustment speed (pricing aggressiveness)
ρ	Speed inertia parameter
β	Penalty coefficient for price fluctuation
Ω	Platform-imposed upper bound on adjustment speed
$\theta_{t}$	Feedback control strength (chaos control parameter) at time t
$H_{t}$	NNormalized permutation-entropy indicator of recent pricing trajectories at time t
$\bar{H}$	Platform’s tolerance threshold for pricing irregularity

## Data Availability

Data are contained within the article.

## Appendix A

### Appendix A.1

**Proof of Proposition** **1.** By the definition of an interior steady state equilibrium and $\omega_{i}^{*}>0$, the equilibrium prices must satisfy $\frac{\partial \pi_{1,t}}{\partial p_{1,t}}=0$, $\frac{\partial \pi_{2,t}}{\partial p_{2,t}}=0$. Under the symmetry condition $p_{1}^{*}=p_{2}^{*}=p^{*}$, the above system reduces to a single linear equation $(1-r)a+bc-(1-r)(2b-d)p^{*}=0$.

When 2b−d>0, this equation admits a unique solution: $p^{*}=\frac{(1-r)a+bc}{\left(1-r)(2b-d\right)}$. Substituting this solution into the demand and profit functions uniquely determines $q^{*}$ and $\pi^{*}$.

Moreover, since prices remain constant at the steady state, it follows that ${\left(p_{1,t+1}-p_{1,t}\right)}^{2}=0$.

The adjustment-speed updating equation therefore reduces to:

$$\omega^{*}=\left(1-\rho \right)\bar{\omega }+\rho \omega^{*}+\alpha \pi^{*}$$

When 0<ρ<1, the above equation admits a unique solution:

$$\omega^{*}=\bar{\omega }+\frac{\alpha }{1-\rho }\pi^{*}$$

Hence, the interior symmetric steady state exists and is unique. Q.E.D. □

### Appendix A.2

**Proof of Corollary** **1.** It follows directly from $\omega^{*}=\bar{\omega }+\frac{\alpha }{1-\rho }\pi^{*}$ that, when $\pi^{*}>0$ holds, we have $\frac{\partial \omega^{*}}{\partial \bar{\omega }}=1>0$, $\frac{\partial \omega^{*}}{\partial \alpha }=\frac{\pi^{*}}{1-\rho }>0$, and $\frac{\partial \omega^{*}}{\partial \rho }=\frac{\alpha \pi^{*}}{{\left(1-\rho \right)}^{2}}>0$. Q.E.D. □

### Appendix A.3

**Proof of Proposition** **2.** A necessary and sufficient condition for the local asymptotic stability of a discrete-time system is that all eigenvalues of the Jacobian matrix lie inside the unit circle, i.e., $\left|\lambda_{i}\right|<1,i=1,2,3,4$. Given 0<ρ<1, it follows that $\left|\lambda_{3}\right|=\left|\lambda_{4}\right|=\rho <1$. Therefore, it suffices to examine $\lambda_{1}$ and $\lambda_{2}$. From $\lambda_{1}=1-(2b-d)(1-r)\omega^{*}$ and $\lambda_{2}=1-(2b+d)(1-r)\omega^{*}$, we obtain:

$$\left|\lambda_{1}\right|<1\Leftrightarrow 0<\omega^{*}<\frac{2}{\left(1-r)(2b-d\right)}$$

$$\left|\lambda_{2}\right|<1\Leftrightarrow 0<\omega^{*}<\frac{2}{\left(1-r)(2b+d\right)}$$

Moreover, since 2b+d>2b−d, it follows that $\frac{2}{\left(1-r)(2b+d\right)}<\frac{2}{\left(1-r)(2b-d\right)}$. Hence, the second condition is more restrictive. Combining the two conditions yields:

$$0<\omega^{*}<\frac{2}{\left(1-r)(2b+d\right)}$$

Q.E.D. □

### Appendix A.4

**Proof of Proposition** **3.** From Proposition 2, a necessary and sufficient condition for local stability of the system is that $0<\omega^{*}<\frac{2}{\left(1-r)(2b+d\right)}$ holds. If, in the absence of regulation $\omega^{u}$, the steady-state adjustment speed exceeds threshold, i.e., $0<\omega^{*}<\frac{2}{\left(1-r)(2b+d\right)}$, the system becomes unstable. In this case, if the platform imposes an upper bound Ω and satisfies $0<\Omega <\frac{2}{\left(1-r)(2b+d\right)}$, the upper bound is both binding and capable of restricting the adjustment speed within the stable region, thereby restoring local asymptotic stability. Q.E.D. □
